# Autonomous Greenhouse Cultivation of Dwarf Tomato: Performance Evaluation of Intelligent Algorithms for Multiple-Sensor Feedback

**DOI:** 10.3390/s25144321

**Published:** 2025-07-10

**Authors:** Stef C. Maree, Pinglin Zhang, Bart M. van Marrewijk, Feije de Zwart, Monique Bijlaard, Silke Hemming

**Affiliations:** Business Unit Greenhouse Horticulture, Wageningen University & Research (WUR), 6708 PD Wageningen, The Netherlands

**Keywords:** computer vision, sensors, dwarf tomato, controlled environment agriculture, autonomous greenhouse control, intelligent algorithms, climate control, plant spacing, data-driven cultivation

## Abstract

Greenhouse horticulture plays an important role globally by producing nutritious fruits and vegetables with high resource use efficiency. Modern greenhouses are large-scale high-tech production factories that are increasingly data-driven, and where climate and irrigation control are gradually becoming more autonomous. This is enabled by technological developments and driven by shortages in skilled labor and the demand for improved resource use efficiency. In the Autonomous Greenhouse Challenge, it has been shown that controlling greenhouse cultivation can be done efficiently with intelligent algorithms. For an optimal strategy, however, it is essential that control algorithms properly account for crop responses, which requires appropriate sensors, reliable data, and accurate models. This paper presents the results of the 4th Autonomous Greenhouse Challenge, in which international teams developed six intelligent algorithms that fully controlled a dwarf tomato cultivation, a crop that is well-suited for robotic harvesting, but for which little prior cultivation data exists. Nevertheless, the analysis of the experiment showed that all teams managed to obtain a profitable strategy, and the best algorithm resulted a production equivalent to 45 kg/m^2^/year, higher than in the commercial practice of high-wire cherry tomato growing. The predominant factor was found to be the much higher plant density that can be achieved in the applied growing system. More difficult challenges were found to be related to measuring crop status to determine the harvest moment. Finally, this experiment shows the potential for novel greenhouse cultivation systems that are inherently well-suited for autonomous control, and results in a unique and rich dataset to support future research.

## 1. Introduction

With a growing population and serious challenges in climate change, there is an increasing need for healthy food produced in a sustainable manner. Greenhouse horticulture plays an important role in producing fruits and vegetables with high land- and water-use efficiency [1,2]. While vegetable production is increasing in area and volume, the number of farms has been declining, resulting in greater cultivation areas per farm and per grower. At the same time, the greenhouse sector aims to reduce its environmental impact by reducing emissions and moving towards circular horticulture [3]. As a result, modern greenhouses are becoming large-scale high-tech production factories that are increasingly data-driven [4]. Sensors and intelligent algorithms are used to intensively monitor and control business and cultivation processes, e.g., for climate management, irrigation, energy usage, internal transportation, sorting, and packaging. This trend has been accelerated by the fast pace of technological developments, such as cloud computing, big data, the Internet of Things (IoT), machine learning, and artificial intelligence (AI) [5]. With the growth of reliable data, the autonomous control of greenhouse climate and irrigation is coming within reach of commercial practice and has been proven to work on an experimental scale [6].

Greenhouse control algorithms have been around for many years [7], and were classically based on proportional–integral–derivative (PID) or model-predictive control (MPC) algorithms. These methods typically require a model of the system, and therefore focus on greenhouse physics by modeling energy balances [8,9]. Such control algorithms are widely implemented in commercial climate computers, in which growers can specify, using setpoints, which climate they wish to achieve in their greenhouse, based on external weather conditions. However, the grower needs experience and skill to determine which indoor climate would be optimal for the crop, and how to exactly achieve this indoor climate, especially since every crop and variety reacts different to certain climates. This makes an integral test of a full cultivation important, as purely simulated experiments never capture the full intricacies of a developing crop. Even in research trials or climate cells, crop responses differ from reality. For autonomous greenhouse control, all components—sensors, actuators, hardware, and software—must work together in a robust manner to steer the crop to be vigorous and productive.

The international competition “Autonomous Greenhouse Challenge” showed the potential of intelligent algorithms in greenhouse control by integrally testing and comparing different control algorithms [10]. In this competition, international teams of multidisciplinary experts developed intelligent algorithms that autonomously control a greenhouse cultivation. The first three editions focused on cucumber crops in 2018 [10], high-wire cherry tomato in 2020 [11], and lettuce in 2022 [12].

The Autonomous Greenhouse Challenge demonstrated that intelligent algorithms can outperform experienced growers in experimental greenhouse trials [10]. The datasets generated from these greenhouse trials provide a unique resource for further research. The dataset of the first challenge on cucumber is publicly available at https://doi.org/10.4121/uuid:e4987a7b-04dd-4c89-9b18-883aad30ba9a, the dataset of the second challenge on cherry tomato at: https://doi.org/10.4121/uuid:88d22c60-21b3-4ea8-90db-20249a5be2a7, and the dataset of the third challenge on lettuce at: https://doi.org/10.4121/21960932.v1. These datasets sprouted the development of many different greenhouse modeling and control algorithms [9,13,14,15,16,17,18,19].

This paper describes the results of the fourth Autonomous Greenhouse Challenge, which took place in the autumn of 2024 and focused on dwarf tomato as a crop. Dwarf tomato is a particularly interesting crop because it requires very little crop handling, and it is harvested in a single instance within a short growth cycle. In that sense, its cultivation is comparable to lettuce, for which almost unmanned production is already in its commercial-ready state. This, plus their small size, makes dwarf tomatoes very suitable for stacked farming and for robotics [20], showing a promise for fully autonomous cherry tomato production. However, very little cultivation data is available about this crop. This currently limits the possibility of control algorithms to make informed decisions about the optimal strategy, making it a challenging crop to control.

The greenhouse trial took place in the autumn of 2024 in six high-tech greenhouse compartments in the Netherlands, as part of the Fourth Autonomous Greenhouse Challenge. During the experiment, for six teams, the goal was to develop intelligent algorithms that had to decide upon climate and crop management strategies. The goal of these algorithms was to optimize net profit by balancing costs and gains. Teams did not disclose the code or the precise inner workings of their algorithms. In this work, we therefore analyze the successful and determining aspects of the control strategies, but do not describe or analyze the inner workings of the algorithms.

The contribution of this paper is two-fold. First, we assess the feasibility, benefits, and limitations of the autonomous cultivation of dwarf tomato. Second, like previous challenges, this experiment provides a rich public dataset of combined climate- and crop data, images, and manual crop measurements, which can boost future research on intelligent algorithms for autonomous control. This public dataset (https://doi.org/10.4121/fa102772-32db-4b30-bace-12f2016722ce.v1) is unique since no other full greenhouse experiments with real autonomous control have been conducted and scientifically been described before for the dwarf tomato. In general, no other autonomous control experiments evaluating algorithms with full real-world data have been described, except the papers on the earlier editions of the challenge.

The remainder of this paper is organized as follows. In Section 2, the experiment setup is described, including the greenhouse compartments, sensors, data platform and cultivation objective. Section 3 describes the results, which are discussed in Section 4. Finally, we conclude in Section 5.

## 2. Materials and Methods

### 2.1. Experiment Setup

Six teams separately developed intelligent algorithms that autonomously controlled six greenhouse compartments with dwarf tomato plants. Five international teams were selected based on their earlier performance in several machine learning tasks. Teams were required to consist of students, technology, or AI researchers and agricultural experts, to stimulate multidisciplinary collaboration. The sixth team, the so-called Reference, was composed of the authors of this work. Teams did not have to disclose the code or precise inner workings of their algorithms.

In the preparation phase, teams got access to a set of training data (publicly available at https://doi.org/10.4121/e1ee9de9-6ce9-4502-a37c-34b5b1372bed.v1). This dataset was collected a year before in a preparatory cultivation in one of the greenhouse compartments, consisting of destructive and non-destructive crop measurements, sensor data, and both canopy and individual crop images. For this dataset, a range of different lighting strategies was applied in order to determine the responsiveness of the crop to light intensity. Additionally, teams had access to the state-of-the-art greenhouse climate simulator Kaspro [8], parametrized on these compartments, and a dwarf tomato crop model.

Starting 1 August 2024, sensors and cameras were installed in the greenhouse compartments. From 15 August 2024, teams could experiment with remotely controlling their greenhouse compartment to test their control system, consisting of sensors and their algorithm. Throughout the entire experiment, teams were not allowed to visit their compartments, so all controls completely relied on sensors and automated actuators. The experiment only involved a single crop cycle, meaning that teams had to develop an algorithm that would be evaluated on its first attempt. This contrasts with the previous edition of the challenge, in which the experiment included two lettuce cultivation cycles [12].

Transplanting happened on 3 September 2024, which marked the start of the experiment. Teams had the opportunity to interfere and to update their algorithm for two more weeks, with the actual crop in the greenhouse. From September 15 until the harvest dates decided by the teams, teams could no longer access their software, so the algorithm was responsible for all operational decisions, including sending messages to re-space the crop and to harvest the end product. Due to the experimental character of the set up, these actions had to be done manually by the greenhouse staff. The experiment ended on 21 November 2024, when the last compartment was harvested. On this day, the total fruit harvest was measured and the winner of the challenge could be announced.

### 2.2. Greenhouse Compartments and Equipment

**Figure 1:**
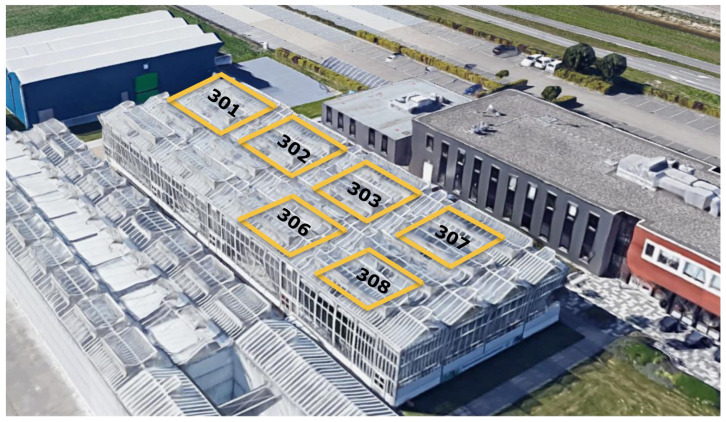
The greenhouse complex in Bleiswijk, The Netherlands (Google Earth image). Each greenhouse compartment is of equal size and has equal base equipment. All compartments have a one side wall at the outer perimeter of the block of greenhouse compartments and one side wall shared with another compartment. The compartments were assigned randomly as follows: Tomatonuts: 3.01, MuGrow: 3.02, Agrifusion: 3.03, Reference: 3.06, IDEAS: 3.07 and Trigger: 3.08.

The six equal greenhouse compartments of 96 m^2^ are located at the research facility of Wageningen University & Research in Bleiswijk, The Netherlands, as shown in Figure 1. These compartments were equipped with standard actuators also available in commercial high-tech greenhouses, which can be controlled via a climate computer (IISI, Hoogendoorn, Vlaardingen, The Netherlands). As a special feature, Hoogendoorn and LetsGrow (Vlaardingen, The Netherlands) created a way to send controls to the greenhouse climate controller through the LetsGrow API. A pipe-rail heating on the floor with a peak capacity of 120 W/m^2^, continuous roof ventilation (ventilation area of 0.3 m^2^ opening per m^2^ greenhouse area, equipped with anti-insect netting with a mesh of 0.40 mm × 0.45 mm), two types of inside moveable screens (LUXOUS 1547 D FR energy screen and OBSCURA 9950 FR W black-out screen, Ludvig Svensson, Kinna, Sweden), and 8 fixtures of VYPR 4i B9F WB dimmable LED lamps (Fluence, Eindhoven, The Netherlands) with a spectrum fixed at Blue 5% − Green 8% − Red 87% + Far-red 8%. The intensity of the LED fixtures were controllable in a continuous range between 20 and 200 µmol/(m^2^ s) and we assume a linear conversion efficiency of 3.2 μmol/J.

The fogging system has an effective maximum capacity of 220 g/(m^2^h), and the ${\mathrm{CO}}_{2}$-dosing system was set to a maximum capacity of 7.5 g/(m^2^h).

The crop was a dwarf cherry tomato (Pick-&-Joy Red Cherry, Vreugdenhil, De Lier, The Netherlands), grown in recycled pots (13 cm, Pöppelmann, Lohne, Germany) with compost-based substrates (Lensli substrates, Bleiswijk, The Netherlands) on tables. Three tables were placed next to each other, each having a size of (1.62 m × 6.12 m and 0.8 m high). Irrigation was supplied via drip irrigation, one dripper of 1.2 L/h per pot with a shot duration fixed to 1 min, meaning that 20 mL of water is supplied per shot. The triggering of these 20 mL watering shots could be controlled by the algorithm. The drain water of all tables was collected. The nutrient recipe was fixed for all compartments, with an EC of 2.8 dS/m. The full nutrient composition is given in Appendix A.

On each table, 200 plants are placed with an initial density of 56 plants per m^2^. Like in modern greenhouses, dwarf tomatoes are grown in varying plant densities, and algorithms could decide to reduce the plant density from 56 to 42, 30, and finally, 20 plants per m^2^. In a commercial greenhouse, plants are spaced the moment the leaves start to overlap with each other. This is to prevent plants from competing for light and growing stretched and uneven. Stretched plants have a tendency to be top loaded with fruits, making them unstable and fall easy, risking production. In this experiment, it was allowed to skip one or more steps, or keep plants at the starting density. Pollination was performed by bumblebees. The hive was opened daily at 10:00 and closed at 14:00, allowing for a sufficient pollination time. The algorithms should make sure that the black-out screen is open during that time frame to allow for sufficient UV light so that the bumblebees can navigate their way back to the hive. Local light pollution regulations further required the closing of the black-out screen between 20:00 and midnight.

In order to have a more complete dataset, at three selected key moments during the cultivation, the fruits of six plants were harvested, sorted by color, counted, and weighed. The results of these measurements were not disclosed and could therefore not be used by the control algorithms. At the end of the cultivation, 50 plants were harvested from the center of the middle tables. Their total fresh weight of ripe fruits was measured. Additionally, the unripe fruits were counted and weighed to be able to estimate potential production.

These final measurements were used to determine the value of the plants, as explained below in the Net Profit calculation.

### 2.3. Sensors and Data Collection

In each greenhouse compartment, standard sensors were made available, comparable to earlier experiments described in [12]. These consist of an outside weather station, obtained weather forecast, indoor climate measurements measured by an aspirated measuring box located in the center of the greenhouse next to the middle table at crop height (temperature, humidity, ${\mathrm{CO}}_{2}$). A standard greenhouse photosynthetic active radiation light intensity (PAR) sensor was placed on the middle table at a fixed height of 0.5 m. Additionally, three pots were equipped with soil sensors (Triple Poseidon WET sensors, Quantified Sensor Technology, Leiden, The Netherlands), capable of measuring the moisture content, electrical conductivity (EC), and temperature in the soil. An overview of all sensor measurements is given in Table A3.

Each greenhouse compartment was further equipped with an Oak-D S2 POE RGBD camera (Luxonis, Littleton, CO, USA). The camera was located facing downwards, about 1.5 m above the growing crop on the center of the middle table. The camera automatically captured hourly images, and allowed for stereo vision and stored a depth image along with the RGB image (3840×2160) and IR image, from which a point cloud could be reconstructed [12]. Images were automatically made available to the algorithms. For irrigation, drain volume, EC and pH were measured.

Furthermore, teams were invited to install additional sensors, as long as these sensors functioned as stand-alone IoT devices, and measured a numeric value (e.g., no additional cameras were allowed). The additional sensor used by the teams are listed in Table A4.

### 2.4. Greenhouse Climate and Crop Control

Prior to the cultivation, teams had to decide on the greenhouse equipment: the Installed Lamp Intensity, and the Number of Screens. These choices determined the fixed costs charged to the teams. Also, the number of spacings applied along the cultivation influenced the fixed costs, as it is reasonable to state that spacing systems allowing more steps are more expensive, either by manual labor costs or by automatic robot handling costs. The parameters available for control during the cultivation are shown in Table A1. These parameters are interpreted on a 5 min basis by the climate computer to control the required actuators in the greenhouse:

- Algorithms could control the air temperature using the Heating Temperature Setpoint and the Ventilation Temperature Setpoint. The direct control of the Minimum Window Position and Heating Pipe Temperature was also possible.
- Increasing the humidity using fogging and reducing the humidity via ventilation were both controlled via the Humidity Deficit Setpoint, with a “deadzone” of 2.5 g/m^3^ before fogging.
- The ${\mathrm{CO}}_{2}$ concentration was controlled using the CO_2_ Concentration Setpoint.
- The LED lamps, as well as the screens, can be directly controlled by providing the Activation Percentage.
- Irrigation was steered by defining the Shot Interval, which is the time in seconds between subsequent shots of 1 min drip irrigation.
- Two crop actions, Plant Density (spacing) and the final Harvest Date, were provided via the same data interface, but executed once a day manually by our greenhouse staff.

When using setpoints for control, the climate computer determined the extent to which the relevant equipment was activated. The states of the greenhouse equipment, such as the window position or heating pipe temperature, were also recorded and could be used in a feedback mechanism (Table A2).

### 2.5. Data Platform

**Figure 2:**
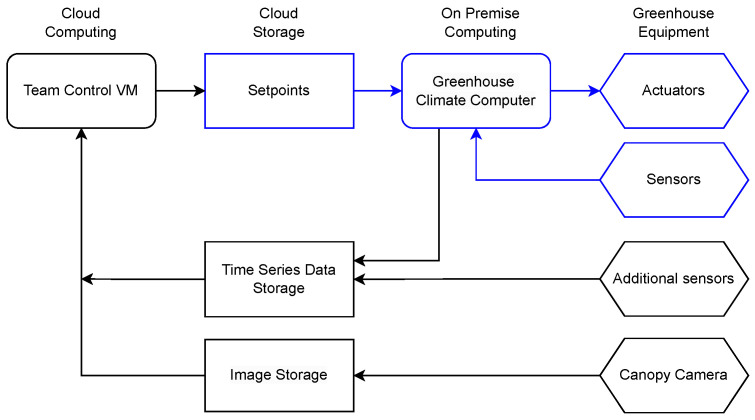
Setup of the greenhouse control system. Arrows indicate the direction of the flow of data. In blue, there is the traditional setup, available in modern greenhouses, where the setpoints are manually defined and stored in the climate computer.

The IT setup is schematically shown in Figure 2. Each team was provided with a Linux-based virtual machine (VM). This VM had a single Nvidia Tesla T4 GPU, 4 AMD EPYC 7V12 virtual CPUs, 28 GB memory and 16 GB of GPU memory. During the experiment, all incoming and outgoing traffic was blocked, except the connection with the cloud storage platform (provided by LetsGrow) and the read-only image storage. LetsGrow provides all access to all the sensor data, as well as the opportunity to write to the setpoints to dedicated data channels via a domain-specific API. These setpoints were then communicated to the greenhouse climate computer, which controlled the actuators.

Routing all traffic through LetsGrow allowed for the careful monitoring of all ongoing and outgoing communication, to ensure that no unfair manual interventions could occur to the control during the experiment.

#### Cultivation Objective: Net Profit

The objective of the cultivation is to maximize the average daily *Net Profit* per $m^{2}$ of greenhouse, i.e., income minus costs,

(1) $$\mathrm{Net}\;\mathrm{Profit}\;[\mathrm{EUR}/m^{2}/\mathrm{day}]=\frac{\mathrm{gains}\;\mathrm{per}\;\mathrm{pot}\;-\;\mathrm{costs}\;\mathrm{per}\;\mathrm{pot}}{\mathrm{average}\;\mathrm{daily}\;\mathrm{area}\;\mathrm{per}\;\mathrm{pot}}=\frac{G-\sum_{d=1}^{D}\left(F+V_{d}\right)\;\cdot \;a_{d}}{\frac{1}{D}\sum_{d=1}^{D}a_{d}},$$

with d=1,…,D counting the days since transplanting and *D* being the Harvest Day chosen by the algorithms. *G* denotes the gains at the end of the cultivation, *F* the daily fixed costs, and $V_{d}$ the daily variable costs. The greenhouse area occupied by a single pot on day *d* is given by $a_{d}=\frac{1}{\delta_{d}}$, and is computed from the Plant Density $\delta_{d}$. Intuitively, in this formula, the net profit per pot is first calculated, which is then divided by the average area occupied per pot.

**Figure 3:**
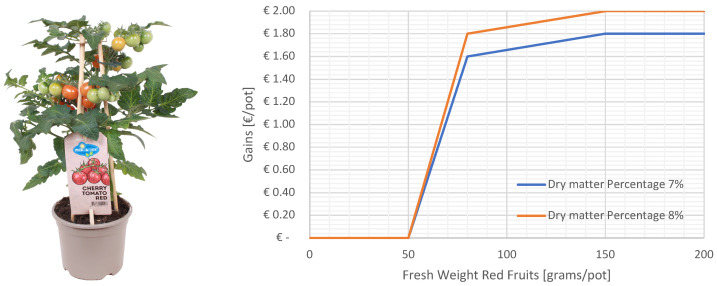
(**Left**): The Pick-and-Joy^®^ concept by Vreugdenhil. The pots are sold to further ripen at home. (**Right**): The price of a pot of dwarf tomato depends on the average fresh fruit weight of the red fruits and the average dry matter percentage of the fruits. When the dry matter percentage is 7% or lower, the low prices are paid (the blue line). When it is 8% or higher, the high price is paid (the orange line). When the dry matter content of the fruits is somewhere in between 7% and 8%, the value is linearly interpolated between the low and high prices.

Plant gains came from the value of the potted plant at the chosen day of harvest. In reality, the value of a plant in this market segment comes from its ornamental value. However, as there is not an unambiguous way to determine the ornamental value from the top view images or quantitative metrics like crop weight, for this challenge, the value was based on the weight of the ripe fruits on the day of harvest. On top of that, the dry matter content of the fruits influenced the price as a higher dry matter content is related to a higher fruit quality. This way of evaluating the results also aligns with the potential market of growing determinant dwarf tomatoes as an efficient cultivation system for cherry tomato production, suitable for full automated production. On the day of harvest, 50 plants are taken from the center of the center table (leaving the two side rows out). From these, all fruits were harvested, sorted by ripeness, counted, weighed, and dried. If less than one third of the fruits was red (rfp<33%), the plants were considered unsellable, P=0. If there were sufficient ripe fruits, the price was then determined by the dry matter percentage (dmp) and fresh weight of the ripe red fruits (ffw), as shown in Figure 3. The plant costs, including the seed, pot, potting soil, and propagation process were set to EUR 0.75 per piece. The gains per pot are then G=P−0.75.

The fixed costs *F* [EUR/m^2^/day] consisted of maintenance and depreciation costs of the greenhouse equipment, which were set to EUR 18.1250 per m^2^ per year (≈0.05 EUR/m^2^/day). Before the start of the trial, teams chose an installed lamp capacity, with a maximum of 200 µmol/(m^2^ s). Depreciation and maintenance of lamps were set to EUR 0.07 per µmol/(m^2^ s) per year. It was up to the algorithms how many spacing changes would be applied, up to three. Each step costs was assumed to EUR 1.50 per m^2^ per year. The resulting formula for the fixed costs per day is then

(2) $$F=0.05+\frac{0.07}{365}\;\cdot \;\mathrm{Installed}\;\mathrm{Lamp}\;\mathrm{Intensity}+\frac{1.50}{365}\;\cdot \;\mathrm{Number}\;\mathrm{Of}\;\mathrm{Spacing}\;\mathrm{Changes}.$$

In this formula, the Installed Lamp Intensity was computed from the maximum Lamp Activation Percentage the algorithms use throughout the trial. The spacing changes are counted via the Plant Density parameter, see Table A1.

The variable costs $V_{d}=L_{d}+H_{d}+C_{d}$ [EUR/m^2^/day] come from three sources: electricity costs of the lamps $L_{d}$, the energy costs for heating $H_{d}$, and the costs for ${\mathrm{CO}}_{2}$ $C_{d}$. To compute the lamp’s power consumption $E_{L}$ in kWh/m^2^, we use the lamp efficiency of 3.2 μmol/J, the maximum intensity of 200 μmol/m^2^, and assume a linear relation between the dimming percentage and the lamp’s power consumption. Since values are recorded at a 5 min interval (12 values per hour), we obtain

(3) $$E_{L}=\frac{\mathrm{LampsActivationPercentage}}{100\%}\;\cdot \;\frac{200\;\mu \mathrm{mol}/m^{2}}{3.2\;\mu \mathrm{mol}/J}/1000/12\;\left[kWh/m^{2}\right].$$

To compute the daily electricity costs $L_{d}$ from $E_{L}$, we considered two electricity tariffs: An on-peak price on workdays (Monday–Friday) between 07:00 and 23:00 of EUR 0.30/kWh, and an off-peak price in the other hours EUR 0.20/kWh.

For a standard greenhouse piperail, which is installed in our compartments, the heating energy consumption $E_{H}$, in kWh per m^2^, can be estimated based on the difference between the heating pipe temperature and the greenhouse air temperature,

(4) $$P_{\mathrm{pipe}}=2\;\cdot \;\max\left(0,\;\mathrm{PipeTemperature}-\mathrm{AirTemperature}\right)\;\left[W/m^{2}\right].$$

These values were only available when the circulation pump was running, and are recorded 12 times per hour. The heating energy consumption is calculated by,

(5) $$E_{H}=\frac{1}{12}\;\cdot \;\frac{P_{\mathrm{pipe}}}{1000}\;\left[W/m^{2}\right]$$

From this formula, The daily heating costs $H_{d}$ were then computed by multiplying by the defined price of EUR 0.09 per kWh. Finally, the ${\mathrm{CO}}_{2}$ usage in kg was calculated by multiplying the positive difference of the ${\mathrm{CO}}_{2}$ Cumulative Dosage Duration, which is recorded in minutes, times 0.125 g per m^2^ per minute, with ${\mathrm{CO}}_{2}$ price set at EUR 0.30 per kg to obtain $C_{d}$.

### 2.6. Secondary Algorithm Evaluation Criteria: Robustness

During the trial, teams did not have access to the virtual machine, their algorithm or code. They had to have robust fallbacks in place in case of missing or delayed data, crashing software or faulty sensors. This is why, as a secondary aim, we analyzed the robustness and autonomy of the algorithms.

The only way for teams to monitor their algorithms was via the public dashboard showing an overview of the last two days of data, as well as summary statistics on the energy and cost components of the cultivation.

If teams, based on the public dashboard, observed a catastrophic failure of their algorithm, they could request an intervention. The organizing team of the challenge approved interventions only when the entire cultivation was at risk—for example, in the case of screens never opening, and there not being any irrigation—or when external factors fully outside of the control of the teams influenced the cultivation.

The cost for interventions was included in the greenhouse challenge as a penalty on the gains per pot of EUR 0.10 at the moment of harvest. For the sake of this paper, we separate these two aims and do not include this penalty.

## 3. Results

### 3.1. Net Profit

The primary goal in this experiment was to maximize Net Profit, as defined in Section Cultivation Objective: Net Profit, for which results are shown in Table 1. All algorithms resulted in a profitable strategy. The algorithm of team IDEAS resulted in the most profitable strategy, with almost twice as much profit compared to the second ranked algorithm of team MuGrow. Both fruit production requirements—the minimum fresh weight of ripe fruits should be at least 50 grams per plant, and at least 33% of all fruits must be ripe—were met by a large margin. In fact, all algorithms chose the harvested moment when over 75% of the fruits were already ripe, and the fresh weight of ripe fruits was over the upper limit of 150 g per pot, up to which additional profits were counted.

**Figure 4:**
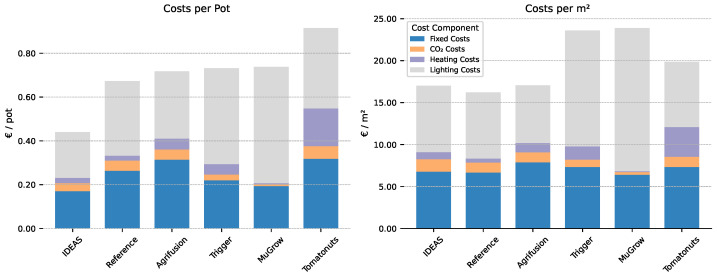
Costs breakdown per intelligent algorithm sorted on the costs per pot (the stacked bar height in the left subplot). Left: the costs per pot, as computed in the net profit. Right: the costs per greenhouse m^2^. Fixed costs per m^2^ vary because teams have chosen a different Installed Lamp Intensity.

In Figure 4, a breakdown of the team costs is shown, per pot, as well as per greenhouse m^2^. This shows that the large net profit of the IDEAS’ algorithm is primarily due to the high plant density, as their costs per m^2^ are similar to the other compartments. The opposite happened for the strategy of Tomatonuts, as their costs per pot are the highest due to a low average plant density. The largest component of the costs is the electricity for the lamps and the fixed costs. High fixed costs further indicate that maximizing production per m^2^ per day is important.

### 3.2. Climate Realization and Energy Usage

**Figure 5:**
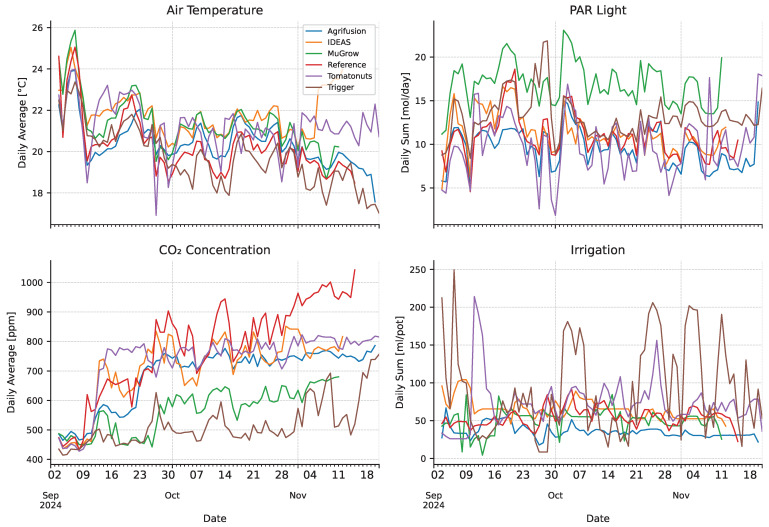
Realized greenhouse inside climate parameters during the cultivation cycle by the different intelligent algorithms. For robustness, the PAR values are calculated from the transmissivity of the screens and cover. Note that different cultivations finish on different days.

Daily climate measurements are shown in Figure 5. The overall averages over the first 70 days of the cultivation (the duration of the shortest cultivation) are shown in Table 2. Large differences can be observed in the resulting strategies. MuGrow applied a high temperature and had by far the largest daily PAR, but had relatively low ${\mathrm{CO}}_{2}$ concentration. Tomatonuts used the most heating energy by a clear margin, while their average air temperature was lower than other teams.

**Figure 6:**
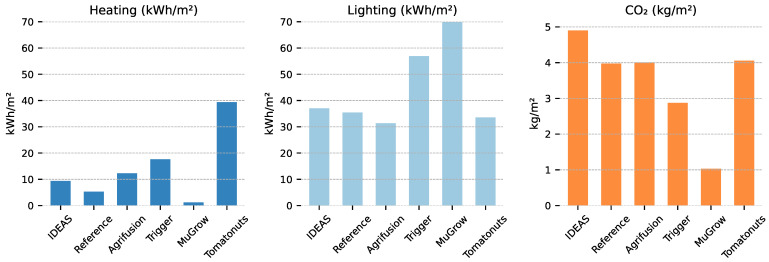
Energy consumption per greenhouse m^2^, calculated as described in Section Cultivation Objective: Net Profit. Algorithms are sorted as in Figure 4, with the left-most algorithm having the lowest costs per pot. Heating energy is converted to kWh (1 kWh = 3.6 MJ) for easier comparison against the electricity input for lighting.

The energy inputs per compartment are shown in Figure 6. Energy inputs are expressed in terms of greenhouse area, instead of per pot, so that the numbers are unaffected by plant spacing, making it easier to compare the efficiency of climate control. For all teams except Tomatonuts, electricity for lighting was the dominating factor. The reference compartment maintained the highest ${\mathrm{CO}}_{2}$ concentration, as shown in Table 2, while in terms of dosage, it is ranked in the middle, suggesting an efficient dosing strategy, with smaller losses due to ventilation. While LEDs are more efficient than traditional HPS lighting, the heat emitted by the lamps, combined with the indirect emission via light absorption, significantly contributes to greenhouse temperature. This explains why MuGrow had a low heating energy input, while still maintaining a high average temperature, compared to the other compartments.

Both Agrifusion and MuGrow applied little irrigation, and their strategies did result in a higher dry matter fraction (Table 1). Trigger applied large amounts of irrigation on sunny days, which could have caused their lower dry matter fraction.

#### Light Use Efficiency

To evaluate the selected cultivation strategy, we calculate the light use efficiency (LUE), defined here as the grams of fresh weight that can be produced at 1 m^2^ per mol of total PAR light in the greenhouse, from both natural light and artificial lighting. IDEAS obtained the highest LUE (10.7 g/mol), while Agrifusion, Reference, and Trigger obtained an LUE of 8.4 g/mol, Tomatonuts 8.2 g/mol, and the lowest was MuGrow with 7.0 g/mol. MuGrow used the most artificial lighting, but this low LUE indicates that the crop was limited by another factor, which could be the ${\mathrm{CO}}_{2}$-concentration or irrigation, which were both low compared to the other teams.

### 3.3. Crop Strategy

**Figure 7:**
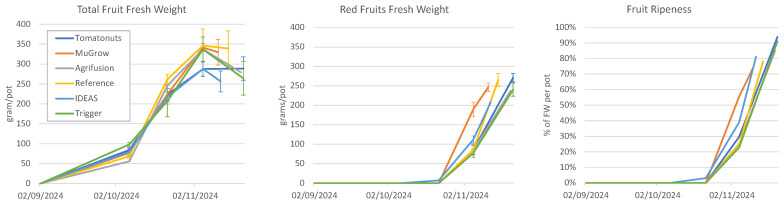
(**Left**): The total fresh fruit weight, measured destructively at 3 moments during the cultivation for all compartments, plus the final harvest moment, which was different per compartment. Error bars indicate the standard error. (**Middle**): Corresponding weight of red ripe fruits, measured on the same plants as the total fresh weight. Error bars indicate the standard error. (**Right**): Fruit ripeness, the amount of red fruit fresh weight as a fraction of the total fruit fresh weight, calculated from the two figures on the left.

Manual crop measurements were taken on 7 October, 22 October, 5 November, and on the day of harvest. On those days, the fruit fresh weight and count were recorded, as shown in Figure 7. A drop in fruit fresh weight at the final harvest date can be observed for most compartments, and while the standard error suggests that this could have occurred due to variation within the crop, it was observed by our greenhouse staff that fruits had fallen off plants.

#### 3.3.1. Harvest Moment

All teams harvested their crop well over the minimal requirements for a profitable product (Table 1). We therefore investigate how much an earlier harvest would have increased their profit.

**Figure 8:**
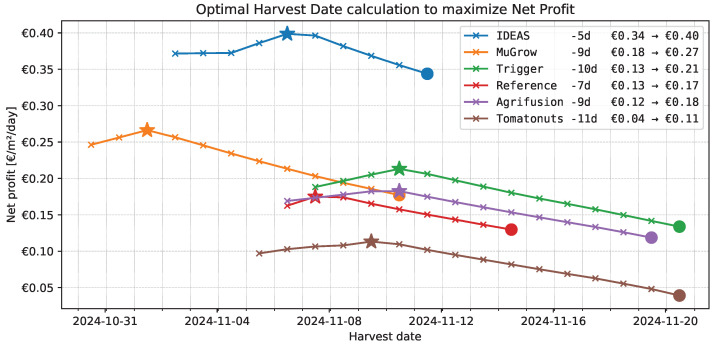
Calculation of the harvest date that maximizes net profit. The circle indicates the real harvest moment, as decided upon by the algorithm. The star indicates the moment in which we calculated that the net profit would have been maximal. The legend indicates how many days earlier this moment was, and what the effect on the net profit would have been. (current → maximal, in EUR/$m^{2}$/day).

Harvesting earlier will reduce costs and allow for more cycles per year. Harvesting too early would, on the other hand, result in too little gains. We calculate the optimal harvest moment as the date on which the Net Profit is maximal. We linearly interpolate the plant measurements that determine the gains—red fruit weight and red fruit percentage—as shown in Figure 7, and assume that the dry matter percentage and the cultivation strategy is otherwise unchanged. Then, we can calculate the optimal harvest moment that would have maximized the profit, as shown in Figure 8. This shows that, in all compartments, the harvest could have been earlier, at least 5 days, up to 11 days. This results in a significant increase in profit, with +EUR 0.06 (+17%) increase for IDEAS up to +EUR 0.09 (+50%) for MuGrow. The ranking remains largely the same, as only the ranks of Agrifusion and Reference are affected.

#### 3.3.2. Plant Spacing

**Figure 9:**
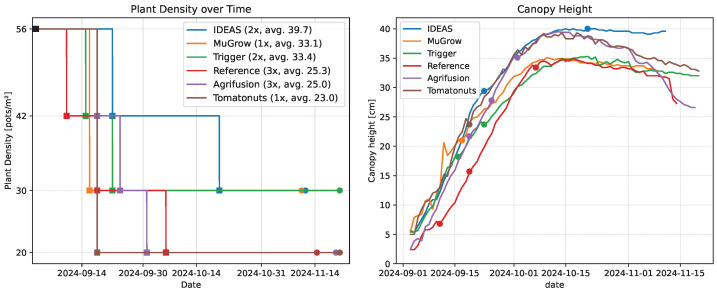
(**Left**): Planting density over time. The black squares indicate a starting plant density of 56 plants/m^2^, which was fixed for all algorithms. Squares indicate the days the algorithms requested a re-spacing. The circle indicates the final harvest day. The legend indicates how many changes have been made, and what the resulting average density was for the entire cultivation. (**Right**): The canopy height, calculated from the RGBD camera images. The canopy height is calculated relative to the top of the pot, as an average of a number of fixed reference points.

The large profit of IDEAS could partially be explained by their high planting density (39.7 pots/m^2^, Table 1), which is visualized over time in Figure 9. Their strategy was to keep a high planting density until very late in the cultivation. The greenhouse staff that applied the re-spacing manually mentioned that this late re-spacing, on 20 October, was difficult because plants were very much intertwined, as they were stretched in order to compete for light.

**Figure 10:**
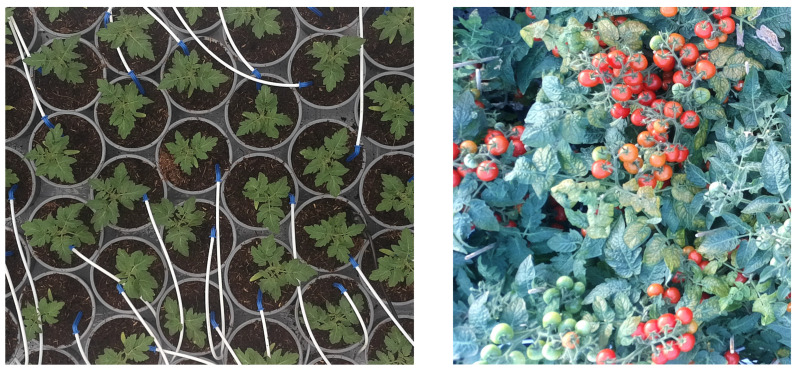
(**Left**): Image (cropped) from the canopy camera on the day after transplanting, Reference compartment. (**Right**): Image (cropped) from the canopy camera on the day before harvest, IDEAS compartment.

As shown in Figure 9, this did result in a canopy height that was slightly higher than the other compartments. Near the end of the cultivation, many plants became top-heavy due to the high plant load, as can be seen from the canopy camera in Figure 10. This caused a decrease in canopy height for all compartments, except IDEAS. In the latter, plants were intertwined, and thereby leaning on top of each other.

#### 3.3.3. Feasibility as a Year-Round Production System

Since the ripening of the fruits happened rather simultaneously, this type of cultivation could be used as a fruit production system, in which all tomatoes are harvested once, after which a new crop cycle can start with fresh plants. A simple calculation using the results in Table 1 shows that the obtained yearly harvest would be 45 kg/m^2^/year for IDEAS, 44 for MuGrow, 41 for Trigger, 35 for Reference, 32 for Tomatonuts, and 30 kg/m^2^/year for Agrifusion. Note that it also affects the algorithms’ ranking, but not the first place.

### 3.4. Autonomy and Robustness

In terms of robustness, all of the algorithms ran stable for over two months without crashing the software. They could handle delayed data, which sometimes occurred, since there are many steps to move data from the sensor to the control virtual machine. Simple but effective measures were taken, such as writing setpoints for the upcoming few days to prevent short-term connection failures. The combination of cloud computing and on-premise control was shown to be effective.

A single intervention was performed during the trial to overrule the decisions made by an algorithm. Trigger, who based their irrigation strategy on a weighing scale, had to intervene when the irrigation completely stopped. Manual instructions were provided upfront about how many pots should be placed on the scale at different spacing levels. However, it did confuse the algorithm such that it stopped irrigating completely.

At the end of the cultivation, both Trigger and Tomatonuts intervened and decided to manually move the final harvest moment forward. Team Trigger argued that their computer vision algorithm did not properly function due to the color differences in the reds of their camera image. Team Tomatonuts did move their harvest date forward by about 10 days, as they feared damaging the fruits due to whiteflies, which were present in the compartment, but not at a rate that our pest experts considered risky. Since the algorithm had no access to scouting data to act upon in the feedback loop, the organizers allowed this intervention.

## 4. Discussion

### 4.1. Net Profit

The results showed that all intelligent algorithms resulted in a profitable strategy. We used reasonable prices during this trial, but did not include all additional costs of operating a greenhouse business, such as harvesting and transport, labor, variable energy prices, insurance or investments.

When the cultivation goal is clearly defined, intelligent algorithms can excel, and this is when these algorithms can outperform experienced growers [11]. By keeping track of costs and resource usage in real-time, every decision can be decided upon its impact towards the cultivation goal. This trial shows that this can be done effectively.

### 4.2. Light Use Efficiency

The light use efficiency is—for all but one team—higher than the 7.7 g/mol reported in the literature [21]. This is a good achievement, but it should be mentioned that this is largely influenced by the high average plant density. The highest light use efficiency obtained by IDEAS can be explained directly by the high planting density they maintained throughout the cultivation. It is worth mentioning that the light spectrum of the LED lamps was fixed for all teams, in order to make light usage comparable. It is possible to optimize photosynthetic efficiency by tuning the spectrum [22,23].

### 4.3. Optimal Harvest Moment

It is interesting that all algorithms decide their harvest moment too late, in terms of the economic objective. Retrospective calculations showed that profits could have been significantly higher if the harvest date had been between 5 and 11 days earlier. It could have been a conscious decision by the teams to delay harvest, as the asymmetric nature of the objective function punishes harvesting too early more. A robust strategy is to harvest a little late, just to be on the safe side. But it is furthermore likely that the problem of determining the right harvest moment is simply hard. The top-view image of the canopy (Figure 10) does not provide a lot of information about the fruit count and ripeness of the lower trusses, especially if the planting density is kept high. In order to determine the fruit ripeness of the lower trusses, additional information is required, for example from crop models, different camera angles or plant sensors, or sensor integration techniques, like applied in lettuce cultivation [12].

### 4.4. Plant Spacing

The largest observable difference between the algorithms was due to plant spacing. It is common sense for pot-plant growers to apply plant spacing over time. In practice, this often happens based on ground coverage or when the leaves of different plants start overlapping, causing competition. For tomatoes, a high planting density increases yield per m^2^, provided it is not so high that it prevents plants from producing enough assimilates to support generative growth, as this can hamper fruit set and reduce fruit size [24,25,26]. This trial confirms that a much higher planting density seems promising. The higher plant density of IDEAS showed a slightly lower fruit count and more small fruits (<6 g). However, the production per greenhouse m^2^ was still higher. This is in line with previous literature [21], which was published shortly before the start of this trial.

On the other hand, undesirable side-effects to high plant density were observed. Multiple compartments showed the yellowing of the lower leaves, and due to stretching, stems were thin, making the plants unstable and difficult to move. For the scope of this experiment, these factors did not play a large role as they were not part of the cultivation objective, but in a commercial setting, this might need to be taken into account.

### 4.5. Feasibility as a Year-Round Production System

The highest yield was 45 kg/m^2^/year, making the dwarf-tomato cultivation system more productive than commercial practice [11,27], in which a typical high-wire cherry tomato production yields 30 kg/m^2^/year in illuminated high-tech greenhouses.

### 4.6. Robustness

It happened a number of times during the trial that data was delayed, or that sensor connections were lost, due to various reasons, caused by both internal or external factors. Control algorithms were able to deal with these in a proper way by having fallback options in place.

Teams argued that it was difficult to use the additional sensors in the autonomous control loop. For some sensors, such as the relative soil permittivity for irrigation or crop feedback signals, the output is difficult to interpret. Other sensors, such as the chlorophyll fluorescence camera, were said to provide valuable insights in, for example, plant stress, but which action should be taken based on this value was not always clear. Future research is needed to enhance knowledge in this area. Another reason for the limited usage of additional sensors is their unknown robustness. In robust autonomous systems, proper backup strategies should be implemented in the case of sensor failure.

### 4.7. Autonomy

In terms of autonomy, in this trial, climate, irrigation, and crop management could be controlled autonomously. Other aspects were not taken into account, such as crop health or pest management. These are difficult to quantify explicitly, especially in terms of their impact on yield or profit.

A robust and healthy crop is essential in cases of unexpected events such as weather extremes, pests and diseases or hardware malfunction, such as clogged irrigation systems. How to integrate these aspects properly into the aim of the intelligent algorithm remains an open research question.

Pest detection was performed with automatic insect detection on sticky traps using computer vision (Trap Eye, Biobest, De Lier, The Netherlands), but scouting on the plants and decision making around biological pest control was still performed manually. In a fully automated system, pest and disease control needs to be integrated.

In this trial, the greenhouse staff regularly inspected the greenhouse, and team members followed their cultivation via public dashboards. Technical maintenance was performed manually, when noticed by local staff, or when anomalies in the data were manually observed. To achieve fully autonomous greenhouse control, these sanity-checks—that have been performed manually—also need to be incorporated into the control feedback loop as well.

## 5. Conclusions

In this paper, we demonstrated that the autonomous greenhouse cultivation of dwarf tomatoes is feasible, especially regarding energy, climate, and irrigation control. Six teams developed intelligent algorithms that autonomously controlled the greenhouse cultivation for over two months. All teams managed to obtain a profitable strategy, and the best algorithm resulted a production equivalent to 45 kg/m^2^/year, higher than in the commercial practice of high-wire cherry tomato growing. The predominant factor was the much higher plant density that can be achieved in the applied growing system. The more difficult challenges are related to measuring crop status to determine the harvest moment. Further improvements may therefore be found in measuring, modeling and controlling crop development, crop resilience, as well as extensions towards pest management and nutrient control. Finally, this experiment shows the potential for novel greenhouse cultivation systems that are inherently well suited for autonomous control, and results in a unique and rich dataset to support future research.

## Figures and Tables

**Table 1:** Results in terms of the cultivations’ primary objective function, Net Profit, underlying parameters to determine the net profit are shown: red fruit fresh weight, red fruit dry weight, red fruits percentage, gains, costs, cultivation duration and average plant density.

Algorithm	Red Fruit Fresh Weight [g/pot]	Red Fruit Dry Weight [%]	Red Fruits [%]	Gains [EUR/pot]	Costs [EUR/pot]	Cultivation Duration [Days]	Avg. Plant Density [pots/m^2^]	Net Profit [EUR/m^2^/day]
IDEAS	219	6.8%	86%	1.05	−0.44	70	39.7	0.34
MuGrow	252	7.3%	76%	1.11	−0.74	70	33.1	0.18
Trigger	268	5.9%	101%	1.05	−0.73	80	33.4	0.13
Reference	275	6.6%	81%	1.05	−0.67	74	25.3	0.13
Agrifusion	258	7.2%	92%	1.09	−0.72	78	25.0	0.12
Tomatonuts	283	6.0%	98%	1.05	−0.91	80	23.0	0.04

**Table 2:** Overall daily climate parameters, averaged per compartment over the first 70 days of the cultivation. Averages are taken until the harvest moment of the first team, for a fair comparison, as the longer cultivations experienced colder and darker days. Temperature and ${\mathrm{CO}}_{2}$ are 24 h averages, PAR, and irrigation are the daily totals.

	Daily Average Temperature [°C]	Daily PAR Sum [mol/day]	Daily Average CO_2_ Concentration [ppm]	Daily Total Irrigation [mL/pot]
Agrifusion	20.6	9.8	678	36.5
IDEAS	21.8	11.3	708	65.8
MuGrow	21.4	17.0	567	50.2
Reference	20.4	11.0	770	55.2
Tomatonuts	21.2	9.6	725	73.6
Trigger	20.0	13.2	503	92.7

**Figure A1:**
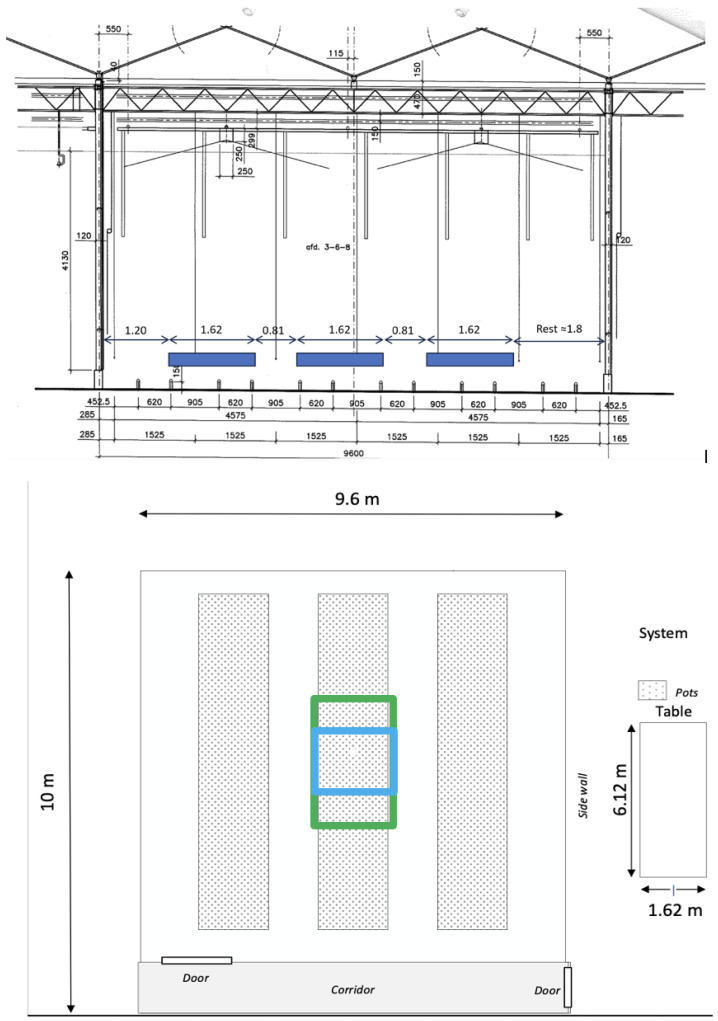
Cross- and top-view sections of one greenhouse experimental compartment in Bleiswijk that will be used in the competition. In the green box, there is the evaluation field (50 pots). In the blue box, there is the camera.

## Data Availability

The complete dataset of the Fourth Autonomous Greenhouse Challenge: Time-series data on realized climate, control states, and weather, together with canopy images, is published online at https://doi.org/10.4121/fa102772-32db-4b30-bace-12f2016722ce.v1. The dataset used by the teams in the preparation of their algorithm is published online at https://doi.org/10.4121/e1ee9de9-6ce9-4502-a37c-34b5b1372bed.v1.

## Appendix A

Each team operates one compartment in a block of eight equal greenhouse compartments. Two out of those eight will be left empty, five will be used by the teams, and one compartment will serve as a reference. A Google Earth image of the block was shown in Figure 1. The setup of the tables in the greenhouse compartments is shown in Figure A1.

The nutrient solution, automatically mixed with an injection unit, was predefined for all teams with an EC of 2.8 dS/m, and nutrient concentrations per liter of 1.1 mmol NH_4_, 12.2 mmol K, 5.6 mmol Ca, 1.8 mmol Mg, 18.7 mmol NO_3_, 1.0 mmol Cl, 2.7 mmol SO_4_, 2.4 mmol H_2_PO_4_, 13.0 μmol Fe, 9.5 μmol Mn, 4.8 μmol Zn, 21.0 μmol B, 0.4 μmol Cu, and 0.3 μmol Mo.

**Table A1:** Greenhouse and crop control parameters. Parameters are updated on a 5 min basis, except for spacing and harvest, which are checked daily. Setpoints are data points sent to the climate computer. For each setpoint, there is a corresponding VIP (Variabel Instel Punt—Dutch for setpoint) which is the actual setpoint the climate computed used. In practice, the setpoint and VIP often match, but influences can affect the value. If there are limits on the change rate of setpoints, to prevent windows or screens from opening and closing too fast, or other influences, these values can be different.

Parameter	Unit	Description
Heating temperature setpoint	°C	If the greenhouse air temperature falls below the this value, the heating pipes are activated.
Minimum pipe temperature	°C	Minimum temperature of the water flowing through the heating pipe. Allows for the direct control of heating without setpoints.
Ventilation temperature setpoint	°C	If the greenhouse air temperature rises above this value, the ventilation windows are opened.
Minimum window position		Forces the windows open, irregardless of the other controls.
${\mathrm{CO}}_{2}$ Concentration setpoint	ppm	If the ${\mathrm{CO}}_{2}$ concentration falls below this value, ${\mathrm{CO}}_{2}$ dosing is activated. The dosing rate is constant, so it is either on or off.
Humidity deficit setpoint	g/m^3^	Ventilation and fogging are both controlled via the humidity deficit (dx) setpoint. There is a “dead zone” of 2.5 g/m^3^ in between the two control actions. The vents are opened if the dx drops below the setpoint; fogging is activated if the dx is more than the dead zone above the setpoint.
Lamp activation percentage	[0, 10–100]%	The lights can either be set off, or dimmed between 10% and 100%.
Energy screen position	[0–100]%	0 meaning the screen is not used.
Black-out screen position	[0–100]%	0 meaning the screen is not used. The screen must be closed at night when the lamps are on, to prevent light pollution.
Irrigation shot interval	Minutes	The interval between subsequent irrigation shots.
Plant density	Pots/m^2^	Allowed values: [56, 42, 30, 20]. Automatic spacing systems are commercially available, but in this small experimental setup, spacing is performed manually by the greenhouse staff.
Harvest day	Day number	The day (Jan 1st = 1) on which all plants should be harvested. Harvest is performed manually by our greenhouse staff.

**Table A2:** Greenhouse actuator state values. Values are measured on a 5 min basis. For faster-changing states, such as ${\mathrm{CO}}_{2}$ dosage and irrigation, cumulative values are calculated by the climate computer and updated on a 5 min basis.

Parameter	Unit	Description
Pipe temperature	°C	Temperature of the water flowing through the heating pipes. A higher temperature means faster heating of the greenhouse, but costs more heating energy. This value is 0 if there is no water flow.
Window position: Lee side	[0–100]%	When the greenhouse temperature is too high, the lee-side first opens.
Window position: Wind side	[0–100]%	If lee-side ventilation does not provide sufficient cooling, the wind side is also opened.
${\mathrm{CO}}_{2}$ Cumulative dosage duration	minutes	Total minutes of ${\mathrm{CO}}_{2}$ dosage. Reset daily.
Irrigation Cumulative water flow duration	Minutes	Total minutes of irrigation. Reset daily.

**Table A3:** Sensor measurement data. Recorded in a 5 min interval. Forecast data is updated a few times per day, and spans the coming 7 days.

Parameter	Unit	Description
Air temperature	°C	
Relative humidity	[0–100]%	
Humidity deficit	g/m^3^	
${\mathrm{CO}}_{2}$ Concentration	ppm	
PAR light intensity	μmol/m^2^s	
Drain volume	liter/10 pots	Because of the data-resolution, this quantity is calculated per 10 pots.
Drain EC	dS/m	
Drain pH	-	
Soil temperature (3×)	°C	
Soil relative permittivity (3×)	-	
Soil bulk-EC (3×)	dS/m	
Outside air temperature	°C	
Outside relative humidity	[0–100]%	
Outside absolute humidity	g/m^3^	
Outside PAR light intensity	μmol/m^2^s	
Global radiation	W/m^2^	
Outside radiation sum	J/cm^2^	Reset daily
Wind speed	m/s	
Wind direction	0–360°	0 = north.
Rain state	0–1	1 = rain, 0 = dry.
Heat emission	W/m^2^	Measured with a pyrgeometer.
Expected outside air temperature	°C	
Expected outside relative humidity	[0–100]%	
Expected global radiation	W/m^2^	
Expected global radiation Sum	J/cm^2^	Radiation sum expected for that day.
Expected degree of cloudiness	1–8	

**Table A4:** Additional sensor measurements, organized per compartment. Each sensor was required to be a stand-alone IoT device.

Team	Parameter	Unit	Sensor Brand and Description
Agrifusion	Plant weight scale	kg	Aranet—plant weight scale
	Microclimate temperature	°C	Aranet—temperature
	Microclimate relative humidity	[0–100]%	Aranet—relative humidity
	Biosignals		Vivent—electrophysiology sensor
MuGrow	Efficiency of photosystem-II	%	Gardin—chlorophyll fluorescence camera
	Photosynthetic capacity	%	Gardin—chlorophyll fluorescence camera
	Energy balance—light saturation	-	Gardin—chlorophyll fluorescence camera
	Electron transport	μmol/m^2^s	Gardin—chlorophyll fluorescence camera
	Actinic—leaf response	-	Gardin—chlorophyll fluorescence camera
	Quantum yield—QE	-	Gardin—chlorophyll Fluorescence camera
Trigger	Plant weight scale	g	Quantified sensor technology—firefly standing scale
	Leaf temperature 1	°C	Quantified sensor technology—firefly infrared thermometer
	Leaf temperature 2	°C	Quantified sensor technology—firefly infrared thermometer
